# A Rare Case of Aortoenteric Graft Erosion Presenting as Candida glabrata Fungemia

**DOI:** 10.1155/2021/9002143

**Published:** 2021-11-16

**Authors:** Muhammad Adeel Samad, Dhaval Patel, Martin Asplund, Diane C. Shih-Della Penna, Yaseen Tomhe

**Affiliations:** Wellspan York Hospital, Department of Surgery, 1001 S. George Street, 2 Main, Surgical Services, York, PA 17403, USA

## Abstract

**Background:**

An aortoenteric fistula (AEF) describes a communication of the aorta or aortic graft with an adjacent loop of the bowel. Aortic graft erosion is a rare complication of abdominal aortic aneurysm repair. We describe a case of a patient presenting with sepsis from Candida glabrata fungemia secondary to aortoenteric erosion without any symptoms or signs of gastrointestinal bleeding. This is a unique case of Candida glabrata fungemia from aortoenteric graft erosion. *Case Summary*. This patient is a 75-year-old male with a history of a prior aortobifemoral bypass graft in 2005. He presented with complaints of right paraspinal pain and chills. He had no symptoms of gastrointestinal bleeding or abdominal pain. His white blood cell count was 25,600/mcl (4,000–11,000/mcL) with left shift. The erythrocyte sedimentation rate was 11 mm/hr (0-38 mm/hr), and C-reactive protein was 95.5 mg/L (<=10.0 mg/L). Blood cultures were obtained and eventually grew Candida glabrata. A computed tomography angiogram (CTA) of abdomen and pelvis demonstrated inflammation surrounding the graft concerning for graft infection with additional inflammatory changes tracking down both femoral limbs. He underwent staged bilateral femoralaxillary bypass followed by the excision of aortobifemoral bypass.

**Conclusion:**

Patients with aortoenteric erosion can present with sepsis in absence of gastrointestinal bleeding. Emergent computed tomography angiogram (CTA) of abdomen and pelvis should be performed to assess for aortic graft erosion or fistula. Empiric treatment with antibiotics should include antifungal agent like micafungin until the final culture is reported. The definite management is an extra anatomic bypass, followed by graft excision.

## 1. Introduction

An aortoenteric fistula (AEF) describes a communication of aorta or aortic graft with an adjacent loop of the bowel [1]. They are typically classified as primary or secondary. Primary AEF exists in the patient's native aorta without prior abdominal aortic aneurysm (AAA) repair. On the other hand, secondary aortoenteric fistula or erosion forms by erosion of the graft into the bowel and is a rare complication of abdominal aortic aneurysm repair [2]. We describe a case of a patient presenting with sepsis from Candida glabrata fungemia secondary to aortoenteric erosion without any symptoms or signs of gastrointestinal bleeding.

## 2. Case Presentation

**Figure 1:**
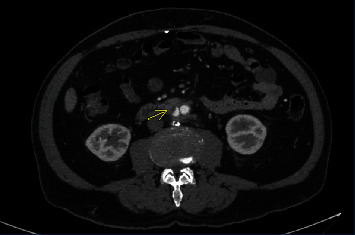
Inflammation around aortobifemoral graft with loss of fat pad between graft and duodenum.

**Figure 2:**
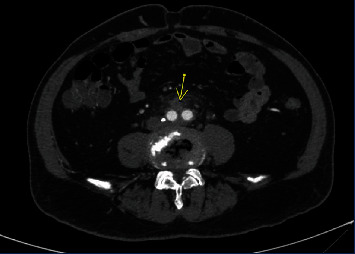
Extension of inflammation distally in both limbs of graft.

A 75-year-old male with history of aortoiliac atherosclerosis requiring aortobifemoral bypass graft at an outside hospital in 2005 presented with complaints of right paraspinal pain for 2 weeks with worsening of pain during the past 24 hours associated with chills. He had no other systemic symptoms of infection, gastrointestinal bleeding, or abdominal pain. His other medical comorbidities were hypertension and hyperlipidemia, and his prior abdominal surgeries included laparoscopic cholecystectomy, right inguinal hernia repair, and open appendectomy. The patient had stable vitals on presentation (afebrile, heart rate: 91 beats/min, blood pressure 142/75 mmHg, respiratory rate: 18 breaths/min, SpO2: 97% on room air). The white blood cell count was 25,600/mcl (4,000–11,000 K/mcL) with left shift. The erythrocyte sedimentation rate was 11 mm/hr (0-38 mm/hr), and C-reactive protein was 95.5 mg/L (<=10.0 mg/L). Lactic acid was 1.8 mmol/L (<2 mmol/L). Blood cultures were obtained and eventually grew Candida glabrata. A computed tomography angiogram (CTA) of abdomen and pelvis was performed as it is the diagnostic modality of choice to assess for aortoenteric fistula. It demonstrated inflammation surrounding the graft concerning for a graft infection with additional inflammatory changes tracking down both femoral limbs (Figures 1 and 2). Loss of fat pad was noted between the duodenum and the graft; however, there was no extraluminal gas. A nonocclusive mural thrombus was present in the proximal right femoral limb with flow distally.

The patient was immediately started on vancomycin and piperacillin/tazobactam. Due to the concern for an infected aortic graft, the decision was made to proceed with a staged extra-anatomic bypass followed by an excision of the infected aortic graft. He underwent bilateral axillary femoral bypass graft with GORE-TEX. Micafungin was added on postoperative day 2 after the blood cultures demonstrated candidemia from Candida glabrata. On postoperative day 4 from the index operation, he was taken for the excision of the aortic graft. Intraoperatively, erosion of the aortic graft was noted into the fourth portion of the duodenum. General surgery was consulted intraoperatively to assist with the dissection of the duodenum. After full mobilization of the graft off the duodenum, a 2 cm defect was identified. The duodenum was closed in 2 layers, using vicryl for full thickness closure of enterotomy followed by Lembert sutures using silk. Both limbs of the grafts were dissected and clamped distally. The graft was excised and explanted from the abdomen. The aortic stump was oversewn. We then performed bilateral groin dissection to remove the distal remnant limbs of the graft and close the common femoral arteriotomy. We noted absence of pulsation in the left axillary-femoral bypass and attempted thrombectomy with a Fogarty catheter with no return of pulsation. The subclavian anastomosis was reexplored, and angulation was identified at the anastomosis which was revised to restore blood flow in the graft.

The patient's postoperative course was complicated by hypotension concerning for postoperative hemorrhage which necessitated a return to the operating room on postoperative day 1 after graft excision. No active bleeding was found, and a small amount of old clotted blood was noted. The patient was transferred to the surgical intensive care unit for continued resuscitation. The cardiac work-up revealed ST elevation in the inferior lead with inferolateral wall motion abnormality noted on transthoracic echocardiogram (TTE). The patient underwent cardiac catherization which showed no significant epicardial coronary stenosis; therefore, no intervention was performed. An upper GI series performed on postoperative day 4 after the graft excision demonstrated no evidence of contrast extravasation from the duodenal repair; therefore, tube feeding was initiated. However, the drainage from both Jackson Pratt (JP) drains was noted to turn a milky color after the initiation of tube feeding. The fluid triglyceride level was 640 mg/dL concerning for chylous ascites. Therefore, the patient was switched to low fat tube feed and slowly transitioned to a low-fat diet which led to the resolution of chylous ascites. Transthoracic echocardiogram and ophthalmoscopy were performed to rule out endocarditis and endophthalmitis in setting of fungemia and both were negative. The final tissue culture of the aortic graft grew Lactobacillus platarum and Candida glabrata. The patient had a slow recovery; however, there were no further complications. He was discharged to acute rehabilitation on postoperative day 18 after the graft excision. He was prescribed 6 weeks of IV micafungin and piperacillin/tazobactam.

The patient was seen in the outpatient clinic where he continued to do well. He had evidence of arterial compromise on the right leg with an ankle brachial index (ABI) of 0.55. The ABI for the left leg was 0.82. He was asymptomatic at this time, and he was treated conservatively with regular follow-up every year using ankle brachial index and pulse volume recordings.

## 3. Discussion

Aortoenteric fistula (AEF) represents one the most challenging complication of abdominal aortic aneurysm repair. Both primary and secondary fistulas are extremely rare. The literature shows that primary AEFs have an incidence of 0.04% to 0.07%, whereas secondary aortoenteric fistulas are a complication of 0.36% to 1.6% of all aortic operations [3].

Secondary aortoenteric fistula can occur months to years after the aortic surgery. The most common theory suggests that the combination of continuous friction between the intestine and the aortic graft and the local inflammation leads to formation of aortoenteric fistula [4]. The duodenum is the most common site for the formation aortoenteric fistula, and a case series of 21 patients showed that the majority of aortoenteric fistulas were duodenal (48%) followed by small intestinal (38%), colonic (10%), and esophageal aortoenteric fistulas (5%) [5]. This has been observed in other case series as well, and our patient also had erosion of the graft into the duodenum. This a likely due to the fixed position of the duodenum and the proximity of the duodenum to the aortic graft.

Secondary aortoenteric fistula is further classified as type 1 or type 2 and has different presentations. Type 1 involves a fistula formation between the aortic graft and the adjacent bowel, commonly involving the proximal aortic suture line. Type 1 typically presents as massive gastrointestinal hemorrhage in 76% of cases. Sepsis and abdominal pain are relatively rare with this type of fistula. Type 2, or a paraprosthetic enteric fistula, involves erosion of the graft into the bowel without formation of a fistula. This generally manifests as sepsis and has been the presenting complaint in 75% of the cases. Bleeding is uncommon but can occur from the edges of the bowel eroded by the aortic graft [6].

One of the largest case series by Schoell et al. comprising of 32 patients revealed that aortoenteric fistula and aortoenteric erosion accounted for 10 and 22 patients, respectively. The most common presentation was bacteremia (53%) followed by sepsis (41%), graft thrombosis (31%), and inguinal abscess (21%). The infection was polymicrobial in 18 patients and monobacterial in 1 patient with E. coli being the most common organism. Candida fungemia was identified in 8 patients; however, individual candida species were not mentioned [7].

Prompt diagnosis of aortoenteric fistula is essential to reduce mortality and morbidity. A case report including 31 patients showed that 17 patients presented with herald bleed while 14 patients presented with massive GI bleeding. The time interval between herald bleed and subsequent massive hemorrhage was unpredictable and ranged from 4 to 92 days [8]. It is essential to recognize patients presenting with herald bleeding so that an intervention can be performed prior to a massive GI hemorrhage. Aortoenteric fistula should be suspected in all the patients with an aortic graft who present with GI bleeding and computed tomography angiogram (CTA) of abdomen, and pelvis with oral contrast should be performed. Computed tomography angiogram (CTA) has variable sensitivity ranging from 40% to 90% for the diagnosis of aortoenteric fistula. The features suspicious for aortoenteric fistula include the presence of ectopic gas, loss of the fat plane between the bowel and the graft, extravasation of aortic contrast material into the enteric lumen, or leakage of enteric contrast material into the paraprosthetic space [9].

The management of aortoenteric fistula or erosion requires excision of the infected graft and revascularization. Placement of an extra anatomic bypass, followed by graft excision, has been the usual treatment [10]. Endovascular aortic stent graft placement and coil embolization provides a safe and effective method of rapidly controlling an aortoenteric fistula with hemorrhage. A case series including 6 patients treated with aortic stent and 1 patient with coil embolization showed that there was immediate cessation of hemorrhage in all 7 cases [11]. However, this is a temporizing measure, and the newly placed prosthetic graft is contaminated with the intestinal flora. The definitive therapy is removal of the aortic graft and repair or resection of the bowel eroded by the aortoenteric fistula after stabilizing the patient and establishing an extra anatomic bypass.

Candida fungemia is rare presentation of aortoenteric erosion. Vegunta et al. reported a case of aortoiliac graft erosion in the duodenum presenting as Candida albicans fungemia and bloody bowel movements [12]. The presentation in our case was unique because the patient presented with signs of sepsis from Candida fungemia without any evidence of gastrointestinal bleeding. Moreover, Candida glabrata fungemia from an aortoenteric erosion has not been reported in the literature previously. Our case also emphasizes that the empiric treatment with antibiotics should include antifungal agent like micafungin until the final culture is reported.

## 4. Conclusion

Patients with aortoenteric erosion can present with sepsis in the absence of gastrointestinal bleeding. Emergent computed tomography angiogram (CTA) of abdomen and pelvis should be performed to assess for aortic graft erosion or fistula. Empiric treatment with antibiotics should include antifungal agent like micafungin until the final culture is reported. Endovascular aortic stent graft placement and coil embolization are safe and effective methods of rapidly controlling hemorrhage from aortoenteric fistula. The definite management is an extra anatomic bypass, followed by graft excision.

## Data Availability

All data is available in the electronic medical record.

## Abbreviations

AEF: Aortoenteric fistula
AAA: Abdominal aortic aneurysm
CTA: Computed tomography angiogram
POD: Postoperative day
TTE: Transthoracic echocardiogram.
